# How Prebiotic Chemistry and Early Life Chose Phosphate

**DOI:** 10.3390/life9010026

**Published:** 2019-03-03

**Authors:** Ziwei Liu, Jean-Christophe Rossi, Robert Pascal

**Affiliations:** 1 UMR5247, CNRS—University of Montpellier—ENSCM, Place E. Bataillon, 34095 Montpellier CEDEX 5, France; zliu@mrc-lmb.cam.ac.uk (Z.L.); jean-christophe.rossi@umontpellier.fr (J.-C.R.); 2 MRC Laboratory of Molecular Biology, Cambridge Biomedical Campus, Cambridge CB2 0QH, UK

**Keywords:** phosphoryl transfer, metabolism, energy currency, mixed anhydride

## Abstract

The very specific thermodynamic instability and kinetic stability of phosphate esters and anhydrides impart them invaluable properties in living organisms in which highly efficient enzyme catalysts compensate for their low intrinsic reactivity. Considering their role in protein biosynthesis, these properties raise a paradox about early stages: How could these species be selected in the absence of enzymes? This review is aimed at demonstrating that considering mixed anhydrides or other species more reactive than esters and anhydrides can help in solving the paradox. The consequences of this approach for chemical evolution and early stages of life are analysed.

## 1. Introduction

Preceding the discovery of the double-helical structure of DNA by more than a decade, the understanding of the metabolic roles of ATP and phosphoryl transfers was an essential step in the disclosure of the foundations of biochemistry by clarifying how energy is distributed and serves as a fuel for the achievement of the different functions of the cell [1]. Biochemists observed that the phosphoryl group is essentially kept within the boundaries of the cell and constantly recycled through the energy-rich intermediates of the metabolism [1]. The determining factor of the properties of phosphate derivatives lies in their negatively charged character, responsible for both their sequestration within compartments delimited by phospholipid membranes as a result of electrostatic forces and for their stability towards hydrolysis and other nucleophilic attacks. Such properties make them suited for their biochemical duties [2]. Westheimer also emphasized “this remarkable combination of thermodynamic instability and kinetic stability” [3]. He expressed how the specific physicochemical properties of these anions are so important for ensuring the different functions played by phosphoryl groups in living organisms: (1) as leaving groups for nucleophilic substitution universal in biology; and (2) as ionized groups useful to conserve metabolites within a compartment having a negatively charged boundary. The values of the pKa for the first ionization (ca. 1–2) [4] are such that a negligible amount of the corresponding biochemicals remains uncharged at physiological pH values. As a result, nucleic acids are conserved within the vesicle as well as many other nucleotide derivatives in such a way that most components of their metabolism remain confined together in a limited volume. From a kinetic perspective, the rates of base-catalysed hydrolysis of phosphoryl derivatives are lowered by electrostatic destabilization of a negatively charged transition state. A noteworthy consequence of this lack of reactivity can be found in the fact that certain phosphate diesters undergo hydrolysis through C-O bond cleavage rather than by that of the P-O bonds [5].

Though their physicochemical properties are adapted to modern biology in which enzymes are able to compensate for the low intrinsic reactivity of negatively charged phosphates mentioned above [3,6], they usually result in sluggish spontaneous phosphoryl transfer reactions, constituting a drawback that probably hampered their selection as reactive intermediates at the chemical stage preceding the evolution of catalytic polymers. In the literature, several authors indeed considered the modern role of ATP in energy exchange as an acquired function and concluded that earlier chemical processes for energy transfer were needed for life to start [7,8].

Building a reasonable scenario for the role of phosphate in the origin and early evolution of life needs therefore to answer two questions, namely: Which kind of reactions could have been prevalent at those stages? What kind of phosphate reactivity could take place spontaneously in the absence of enzymes? These questions become even more crucial taking into account the fact that early membranes, probably more permeable than those based on phospholipids, were less efficient in sequestrating anionic metabolites. No obvious selective advantage of derivatives involving phosphoryl groups can therefore be foreseen at the prebiotic stage when no efficient coded catalyst could compensate for the low intrinsic rates of most phosphoryl transfers. Low-valence phosphorus derivatives have been proposed as an alternative to provide a higher reactivity [9,10,11,12]. Without prejudice to the actual relevance of this attractive possibility, our work is aimed at determining which chemical pathways could have been critical for the introduction of phosphate anhydrides and esters as intermediates in early biochemistry. Our main tenet is related to the importance of mixed anhydrides that can be formed from phosphates and high-energy carboxylic acid derivatives and that may have contributed to the distribution of energy in early metabolisms. The fact that phosphates could play a role in chemical and early biochemical evolution could therefore be related to a very peculiar chemistry having a limited relationship to the usual biochemical role of phosphate derivatives. Though the importance of phosphate chemistry in the structure and stability of biomolecules and biopolymers will be mentioned, this review is mainly focused on reactivity issues related to phosphoryl transfers and their potential contribution to the distribution of energy in protometabolisms or early biological metabolisms.

## 2. Phosphoryl Transfer Pathways

### 2.1. Phosphate Esters

**Figure 1:**
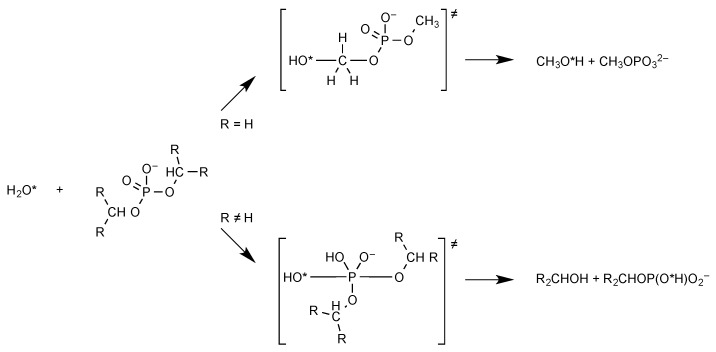
Hydrolysis of phosphate diesters. Nucleophilic attack can take place at carbon or phosphorus depending on the degree of substitution at carbon.

At moderate pH, chemical transformations at the phosphorus centre of phosphoryl groups usually take place from the monoanion. However, there is a profound difference depending on the degree of substitution at phosphorus. The situation is clearly illustrated by the difference in reactivity between diesters and monoesters. Phosphodiesters are highly stable to hydrolysis largely because the presence of a negative charge at moderate and alkaline pH values constitutes a barrier towards nucleophilic attack, which can be appreciated by considering the alkaline hydrolysis of the simplest model of phosphodiester, dimethyl phosphate (Figure 1). In an unexpected way, this reaction takes place through a nucleophilic substitution at carbon rather than at phosphorus [5]. The P-O bond of phosphodiesters is therefore almost unreactive towards hydrolysis at physiological pH values explaining why a phosphodiester backbone could have been selected by evolution for the long-term storage of information in DNA [13]. We can conclude that the relevance of phosphate diesters to the origin or the early developments of life rather lies in their chemical resistance than in their reactivity. Making this reaction compatible with a protometabolism time scale would require the lifetime of phosphodiesters to be reduced from tens of million years into days [14]. Such values of rate enhancement exceeding 10^8^ are only accessible through catalysis by enzymes. Simple chemical catalysts could hardly reach that efficiency except intramolecular reactions in which the proximity of reacting groups can compensate for the kinetic barriers [15]. The well-known instability of RNA compared to that of DNA precisely lies in the presence of a hydroxyl group at the ribose 2′-position capable of provoking a cleavage of the internucleotidic linkage intramolecularly. This observation supports the importance of intramolecular processes before the advent of enzymes [16,17,18].

**Figure 2:**
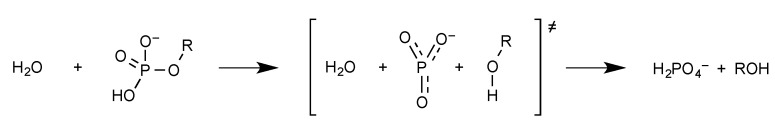
The dissociative pathway of phosphoryl transfer in the hydrolysis of phosphate monoesters. A metaphosphate ion (PO_3_^−^) intermediate or at least resonance stabilization at the transition state is involved in the reaction.

Monoesters such as methyl phosphate [5] behave in a completely different way and are much less stable than diesters at neutral and mildly acidic pH values. In contrast with the reaction of phosphodiesters corresponding to an associative mechanism, monoesters are cleaved through dissociative transition states resembling the resonance-stabilized metaphosphate ion (PO_3_^−^, Figure 2) [19,20]. There has been a long-lasting debate on the actual lifetime of the metaphosphate ion, which may not be sufficient for it to be considered as a true intermediate and the dissociative nature of the reaction pathway has been disputed on theoretical grounds [21]. Anyway, it can be acknowledged that resonance stabilization plays a role at the transition state so that the hydrolysis of phosphate monoesters is ca. 6 orders of magnitude faster than the corresponding reaction of diesters assessed using substrates unable to undergo substitution at carbon [13].

In spite of a faster reaction, phosphate mono-alkyl esters still present lifetimes (measured in tenth of years at moderate values of pH and temperature [13]) incompatible with a role of reactive intermediates of a metabolism. However, this stability is the basis of their role of constituents of basic structures of the cell such as phosphatidic acids as components of the membranes and other metabolites bearing an anionic charge, allowing them to remain sequestrated within the boundaries of the cell [3].

### 2.2. Phosphate Anhydrides

**Figure 3:**
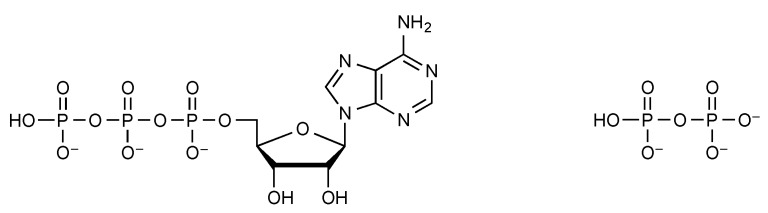
ATP and pyrophosphate have been proposed as prebiotic energy currencies, in spite of the kinetic barrier hindering their reactions with nucleophiles at moderate pH values at which they are negatively charged.

The presence of a much better leaving group in phosphate anhydrides tends to increases their reactivity. However, it must be taken into account that this effect is offset in anhydrides such pyrophosphate and ATP (Figure 3) by the presence of 3 or 4 negative charges at moderate pH values, rendering these activated species spontaneously almost non-reactive towards nucleophilic attack since it hinders the development of more negatively charged transition states. Therefore, most reactions of these high-energy intermediates require catalysis to take place at rates compatible with the time scale of a metabolism. In the living world, this limited reactivity results in a kinetic stabilization and the reactivity of ATP can be orientated towards specific paths by enzymes like kinases, which are responsible for many cellular functions and for the role of ATP as an energy currency. With regards to the origin of life, an advantage can hardly be expected from this exceedingly limited reactivity due to the lack of selective catalysts for many phosphorylation processes. Pyrophosphate and more generally polyphosphates would suffer from a similar lack of reactivity making their involvement in prebiotic chemistry and early biological evolution questionable even though that contribution to the chemistry of the origins of life has been proposed in many instances [22,23,24,25,26,27,28,29,30]. Independently of the limitations to their possible role of energy currency, a similar difficulty related to the lack of enzyme catalysts has been raised when considering the biochemical use of nucleoside triphosphates as activated monomers for RNA oligomerization [31].

### 2.3. Phosphate Mixed Anhydrides

Acyl phosphates are among the more potent activated biochemicals [32]. Values of their free energy of hydrolysis at pH 7 (Δ*G*°’) reach −43 kJ mol^−1^ for acetyl phosphate [33]. By contrast with ATP and pyrophosphate, acyl phosphates and acyl adenylates bear a single negative charge at mildly acidic pH values and benefit to a much lesser degree from the kinetic stabilization that inhibits the increase of negative charge at the transition state of the reactions with nucleophiles including water and hydroxide ion. This limitation is likely to be even less stringent for mixed anhydrides of inorganic phosphate that can be cleaved through a dissociative mechanism, in which resonance stabilization occurring within a transition state resembling metaphosphate ion replaces the interaction with the nucleophile as the main driving force [34].

**Figure 4:**
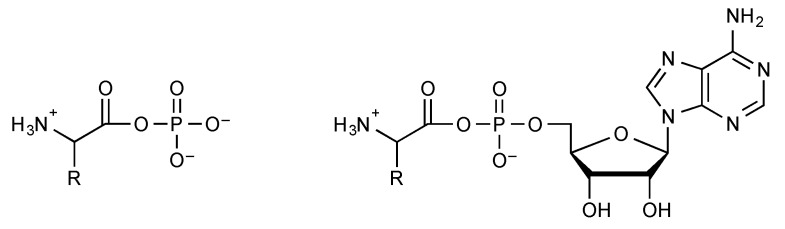
Amino acyl phosphates and aminoacyl adenylates are highly activated biochemicals.

**Figure 5:**
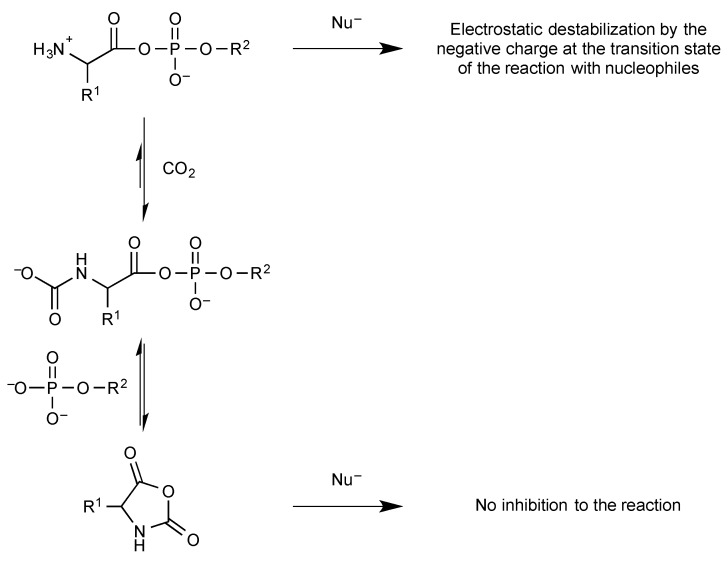
Phosphate esters mixed anhydrides suffer from stabilization against reaction with nucleophiles as other phosphate derivatives. The pathway involving *N*-carboxyanhydrides (NCAs) as intermediates must be taken into consideration as soon as CO_2_ is present in the atmosphere, even at low levels.

Indeed, aminoacyl phosphates (Figure 4) were shown to undergo a cleavage of the P-O bond and constitute efficient phosphorylating agents [35]. Mixed anhydrides with phosphate monoesters like acyl adenylates remain hydrolytically unstable and susceptible to spontaneously undergoing reactions with other nucleophiles at the carboxyl moiety. These mixed anhydrides can be formed as intermediates in the reactions of various acyl donors including activated esters [36,37,38], thioesters [39,40], or anhydrides [37,41]. The biochemically essential acetyl phosphate can for instance be formed photochemically by oxidation of the thioacid [42]. A similar reaction of thioacetate has recently been reported to occur in limited yields in a hydrothermal context [43] without mention of the possibility of photo-oxidation [42]. Amino acyl adenylates (Figure 4) deserve a particular mention because of their role in protein biosynthesis. Their degree of activation has been assessed in the case of Tyr-AMP to a value of Δ*G*°’ = −70 kJ mol^−1^ [44] much higher than that observed for simple acyl phosphates and that exceeds the values observed for the main intermediates of energy metabolism including phosphoenol pyruvate. Amino acyl adenylates are formed biochemically by the reaction of amino acids with ATP. However, because of the endergonic character of the reaction, amino acyl adenylates usually remain sequestrated in the active site of aminoacyl-tRNA synthetases, the enzymes that are responsible for their formation from ATP and for the further aminoacylation of tRNA [45]. Abiotically, the formation of mixed anhydrides through a similar reaction of phosphate anhydrides with unprotected amino acids can therefore be considered as unlikely for thermodynamic reasons in addition to the sluggish kinetic availability of ATP due to its multiple negative charges. Therefore, science has to solve the paradox of the initial formation of aminoacyl-adenylates required for the evolution of translation but impossible from ATP without enzymes. That paradox requires the occurrence of alternative pathways [46,47]. This possibility has experimentally been supported by the observation that two categories of prebiotically plausible activated derivatives of α-amino acids undergo spontaneous conversion into aminoacyl adenylates or related mixed anhydrides. 5(4*H*)-Oxazolones, formed as a result of the strong activation of acylated amino acids or peptides, have demonstrated an ability to be converted spontaneously into mixed anhydrides in the presence of inorganic phosphate or phosphate esters [48,49]. These reactions yield peptidyl- or acyl-substituted products but derivatives with a free amino group can be obtained directly by the analogous reaction of amino acids *N*-carboxyanhydrides (NCAs) [35,50,51]. NCAs have been proposed as plausible activated forms of amino acids under prebiotic conditions and several potential pathways are available for their formation [46,52,53,54,55]. It is worth emphasizing that any reaction involving phosphate as well as its monoesters as nucleophiles and activated carboxylic acids would be facilitated rather than kinetically inhibited by phosphate negative charges, which avoids the need for catalysis for an abiotic process. A potential role for these intermediates in the chemical processes associated with the development of life is therefore highly likely, provided that carboxylic acid activation into high-energy intermediates is possible in that environment. Since NCAs are formed rapidly from most other forms of activated α-amino acids having a free amino group in aqueous media containing carbon dioxide [52], the prebiotic relevance of phosphate mixed anhydrides of amino acids should be recognized provided that phosphate is available. However, the fast reverse reaction of carbon dioxide also prevails from phosphate mixed anhydrides of α-amino acids, which are converted back into NCAs rapidly [48,56]. Though present to a lesser degree than in polyphosphates like ATP or pyrophosphate, the negative charge of phosphate esters mixed anhydrides reduces their reactivity with nucleophiles so that the reaction pathway may involve a prior conversion into neutral (and therefore highly reactive) NCAs rather than a direct reaction of amino acid phosphate anhydrides (Figure 5). Accordingly, the polymerization of aminoacyl adenylates into peptides takes place through the NCA pathway [48] rather than from a direct polymerization as proposed earlier [57]. 

The role of phosphate mixed anhydrides in the development of life should therefore be analysed by taking into account two counteracting factors: (1) a less important kinetic stabilization by negative charges as compared to polyphosphates; and (2) but a relative stabilization compared to neutral highly reactive activated acyl precursors. Generally no kinetic advantage is therefore to be expected from a reaction of mixed anhydrides compared to activated acyl precursors as in the case of the formation of peptides in which the fast polymerization of NCAs competes favourably with the polymerization of mixed anhydrides [48]. However, in some cases their reactivity could be advantageous as probably in the case of the aminoacylation of the 3′(2′)-end of RNA for which NCA proved to be inefficient [58]. Examples of an advantageous role of mixed anhydrides have been observed from their involvement as intermediates undergoing a fast intramolecular acyl transfer as in the formation of esters with ribonucleotides [50,56,59,60]. Phosphate moieties could indeed act as handles capable of reacting with activated acyl moieties and then to intramolecularly transfer the acyl group to a poor nucleophile thanks to the entropic advantage of intramolecular processes [16,61]. This property provides a rationale for the selection of mixed anhydrides in the evolutionary process. On the other hand, the easy conversion of activated acyl derivatives including those of α-amino acids into phosphate mixed anhydrides might be considered as an early example of how free energy could be exchanged between the chemistries of α-amino acids and that of nucleotides predating the role of ATP as an energy currency [46,47]. Thioesters constitute other activated acyl derivatives that yield phosphate mixed anhydrides by interaction with phosphate. Pathways leading to their formation from carbon chemistry have been proposed [62]. This contribution could be important for many thioesters with the exception of α-amino acid thioesters that are rapidly converted into NCAs in the presence of CO_2_ or bicarbonate [52] so that their reactivity cannot be considered as different from that of other activated α-amino acid derivatives.

### 2.4. Phosphoramidates

**Figure 6:**
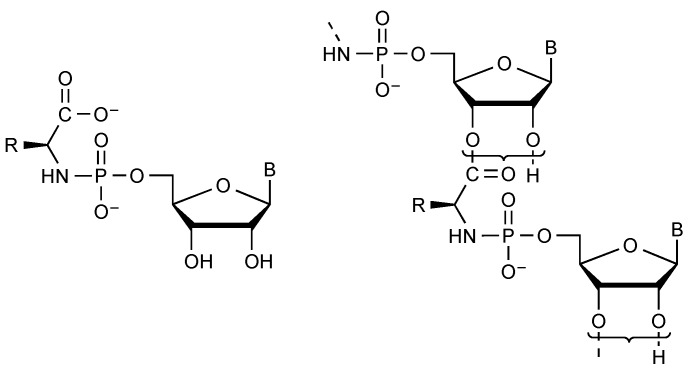
Phosphoramidate derivatives of amino acids and the ester- and phosphoramidate-based structures constituting the basis of prebiotically plausible copolymers.

The chemical interaction of free α-amino acids with activated phosphates can also yield phosphoramidate derivatives by nucleophilic reaction of the amino group (Figure 6). By contrast with the behaviour of carboxylic acid derivatives, phosphoramidates are more reactive than phosphate esters and correspond to an activated state of phosphate, which has been illustrated by the ability of some of them to behave as polymerase substrates for the synthesis of DNA [63,64,65,66,67,68]. In addition to that ability in enzyme reactions, *N*-phosphoryl amino acids proved to be capable of yielding both phosphate esters and polypeptides through spontaneous reactions in aqueous solution [69,70,71]. Lastly an intermediate role has been proposed for phosphoramidates in the polymerization of amino acid promoted by EDC (1-ethyl-3-(3-dimethylaminopropyl)-carbodiimide) in the presence of nucleotides [72,73]. A contribution of phosphoramidates to prebiotic chemistry and early biochemistry can therefore be considered as highly likely as soon as powerful activating agents were present. Interestingly, activating agents based on phosphoramidate moieties have been proposed in an origin of life context [74,75,76,77]. In addition to being involved as intermediates in the formation of biopolymers, it is worthy to note that chemical ligations as well as template-directed polymerization proved to proceed more easily using modified nucleotides bearing an amine nucleophile instead of the the 3′-hydroxyl group yielding phosphoramidate linkages owing to the increased nucleophilic power of amines compared to alcohols [78,79,80,81,82]. The facilitated nucleotide polymerization has allowed major studies of the replication process proceeding in the absence of enzymes [31,83,84,85]. It could also be considered as a basis for the formation of mixed structures [60,86] involving both amino acids and nucleotides bound through ester and phosphoramidate linkage with an unexpected lifetime for aminoacyl esters (Figure 6) [60].

## 3. Which Phosphate Derivatives Could Play A Role as Early Energy Currencies?

The availability of free energy is crucial for self-organization to maintain a system in a far from equilibrium state [87]. However, this energy must not be dissipated directly through a linear spontaneous process in order that work can be carried out. In other words, as Eschenmoser [88,89] emphasized using a different terminology, the chemical environment must be held far from equilibrium by kinetic barriers. From this point of view, the kinetic stability of ATP makes it a unique component of metabolism. ATP is well known for its ability to act as an energy currency that it is constantly synthesized and used up by hydrolysis into ADP and inorganic phosphate [1]. An open question with respect to early metabolism is related to the probable inability of ATP to play this role and, consequently, to the possible existence of others chemicals acting as substitutes. In earlier reports, a body of evidence was gathered to support the idea that ATP could not be involved as an energy source for the development of translation [46,47]. This conclusion was mainly based on the observation that there is no chemical (non-enzymatic) path available for the conversion of ATP into amino acid adenylates for both thermodynamic and kinetic reasons. Namely, the free energy potential of ATP is unable to afford significant concentrations of adenylates at equilibrium with amino acids in the presence of ATP and the thermodynamically favourable reverse reactions yielding ATP from adenylates and pyrophosphate do not spontaneously take place and require the presence of enzymes. 

**Figure 7:**
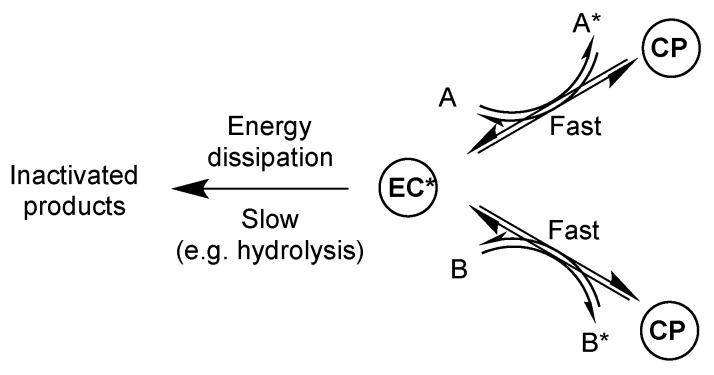
An energy currency (activated form EC*) formed from a currency precursor (CP) requires a high free energy potential and pathways available to transfer energy between different processes faster than the dissipation of energy. Energy currencies must therefore comply with kinetic and thermodynamic requirements.

Considering the properties required for a chemical species to act as an energy currency (Figure 7) should be helpful in identifying alternatives. A first requirement corresponds to a far from equilibrium state meaning that the thermodynamic potential of the currency makes it able to dissipate energy in the environment. Potential energy currencies can therefore be considered on the basis of their thermodynamic potential (see Table 1). However, dissipation must be hindered by kinetic barriers so that the energy currency can act as an activating agent able to produce work by delivering energy to other components of the system (Figure 7). This second condition, which could seem somewhat contradictory with the preceding one, corresponds to the need for kinetic stability of the potential candidate that must be able to transfer its energy to a recipient chemical system with rates faster than, or at least competing with, those at which its potential is dissipated in the environment through breakdown processes (e.g., by direct hydrolysis). As far as non-living systems are concerned, limited possibilities of *selective* catalytic pathways are available to make reactions rates consistent with the time scale of the system *without increasing those of dissipation pathways*. In spite of the fact that they have been proposed as early analogues of ATP, polyphosphates including pyrophosphate fail to fulfil that latter kinetic requirement. Therefore, both the above-mentioned inability to activate amino acids into adenylates and a poor spontaneous reactivity can be considered as indications that pyrophosphate and other polyphosphates could hardly play a role in energy transduction in early metabolisms unless efficient catalytic pathways for the transfer of their energy are found in the future. Anhydrides bearing less negative charges would react faster, which supports a potential role of carboxylic-phosphoric mixed anhydrides. From an energy perspective, a thermodynamic potential sufficient to allow for the formation of aminoacyl adenylates was required for the emergence of translation and more precisely for the evolution of aminoacyl-tRNA synthetases (aaRS) that use amino acids activated as adenylates. Amino acid *N*-carboxy anhydrides (NCAs) have been proposed as essential intermediates in this context [46,47,90]. NCAs were identified as reagents capable of providing adenylates without requiring catalysis by enzymes [50,51]. The value of their free energy of hydrolysis at pH 7 (Δ*G*°’ = ca. −60 kJ mol^−1^ [46]) associated with a spontaneous reaction with phosphate and phosphate mono-esters makes them likely precursors of mixed anhydrides, including adenylates. The inability of ATP to provide adenylates in a similar way shows that another reagent played its role or that no reagent played the role of universal energy currency. However, some of the species of Table 1 having a high potential, could be formed abiotically or at least without requiring catalysis, some of them, including acetyl phosphate (as other acyl and aminoacyl phosphates) and carbamyl phosphate, indeed still play a role in biochemistry. They could be considered as possible alternative energy shuttles between different systems, notably able to yield mixed anhydrides required for different metabolic functions in early living organisms, without reaching the status of universal energy currency as ATP in evolved living system.

## 4. The Question of Prebiotic Phosphorylation

The abiotic formation of phosphorylated metabolites is a central issue in prebiotic chemistry and comprehensive reviews dealing with this question and providing a list of reagents relevant to the origin of life context have been published [92,93]. The possibility of a contribution of phosphates to prebiotic chemistry and the origin of life should have been limited by the availability of phosphate or other phosphorus containing intermediates (including low valence derivatives). Solution phosphorylation would for instance be limited by the solubility of phosphate, which is strongly reduced in the presence of di- or tri-valent cations [30]. As these ions were likely present in the environment on the prebiotic Earth, the low content of phosphate in solution should be considered as unfavourable to phosphorylation. However, the low availability of phosphate in an ocean could be compensated in some cases by the favourable effect of cations on the phosphorylation reaction. A phosphorylation process involving cyanate as an activating agent and precipitated apatite was reported as a realistic pathway in prebiotic chemistry, which means that the reaction can take place on the surface of the solid [23]. The activation of inorganic phosphate can take place by reaction with energy-rich chemicals (Table 2). 

**Figure 8:**
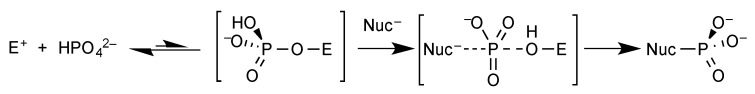
Phosphorylation can by promoted by electrophilic activating agent capable of generation an intermediate capable of transferring the phosphoryl group to an acceptor nucleophile through a metaphosphate or, at least, resonance-stabilized intermediate.

Cyanamide dimer [95] or cyanate [96,97] are able to promote the formation of reactive adducts with inorganic phosphate that subsequently act as phosphoryl donors, very probably through a dissociative pathway involving a resonance-stabilized transition state (Figure 8). 

**Figure 9:**
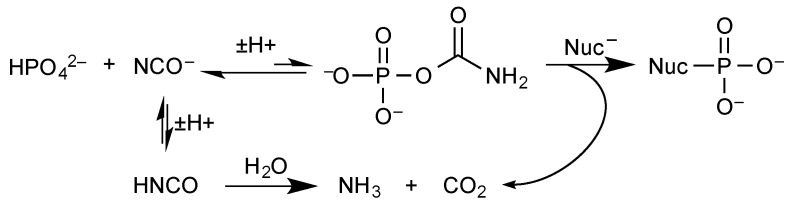
Cyanate-promoted phosphorylation through a carbamyl phosphate intermediate.

The reaction of cyanate is well-documented, yielding carbamyl phosphate as a transient species upon reaction with inorganic phosphate [96,97]. Then the intermediate decays either through hydrolysis yielding eventually CO_2_ and NH_3_ or through an elimination pathway specific of the mono-anion [98,99] and reverting cyanate (Figure 9). 

The overall process constitutes a catalytic pathway of hydrolysis of cyanate quite similar to that observed for carbonate and dicarboxylic acids, initially reported to involve general acid catalysis [100], but later proven to actually correspond to nucleophilic catalysis [101]. Carbamyl phosphate can also be prepared photochemically from Fe(CN)_6_^3−^ and is able to promote the formation of ATP or acetyl phosphate [102,103,104]. 

The most important limitation for the formation of phosphate monoesters in diluted aqueous solution lies in the usual low selectivity of the reaction of alcohols compared to that of water in large excess that outcompetes that of diluted substrates. A very attractive possibility to solve this issue lies in the use of chemical catalysis. The condensation of aldehydes with diamidophosphate provides a pathway to regioselectively phosphorylate glycoaldehyde and other aldoses very efficiently through an induced intramolecular pathway [74,75,76,77]. Phosphoryl transfer in a supramolecular environment has also been useful to promote selective phosphorylation [105,106]. 

Performing the reaction under dehydrating conditions under the effect of heat has been considered as another possibility to avoid dilution in aqueous solution. Heating mixtures of reagents to temperatures above 80 °C in the presence of ammonium formate [27], or formamide [107] proved to be efficient though the regioselectivity was limited. Since its first mention [108], urea has been used in many instances to perform phosphorylation of nucleotides [109] or long-chain alcohols [110] under the effect of heat. It is worth noting that reactions in urea-inorganic phosphate mixtures proceed faster than with the other additives. No definitive answer has been given to the actual pathway through which urea promotes phosphorylation. Phosphoramidate or carbamyl phosphate intermediates [108] have been mentioned as possible actual phosphorylating species. An activated intermediate of unknown nature has also been proposed [109]. Other explanations involve nucleophilic [93,111] or acid–base [112] catalyses. A more likely explanation has been proposed [113] that takes into account the easy breakdown of urea into cyanate at high temperature [114]. This ability of urea independently accounts for the formation of amino acids *N*-carboxyanhydrides in hot aqueous solutions from urea [55]. It would however mean that the activity of urea for promoting phosphorylation is the result of a stoichiometric rather than catalytic reaction involving cyanate as an activating intermediate and carbamyl phosphate as the actual phosphorylating agent (Figure 9). 

## 5. Conclusions

This review focuses on the specific features of phosphoryl group reactivity that raise constraints on the prebiotic and early biochemical pathways involved in the origin of life and its early developments. The charge of phosphate moieties constituted a determining advantage for sequestrating substrates as soon as phospholipids, fatty acids or other negatively charged amphiphiles were present and able to form membrane-delimited compartments. Another essential biochemical consequence of this charge is the resistance of phosphate moieties to nucleophilic reactions and most notably to base-catalysed hydrolysis that is hindered by repulsive electrostatic interactions at the transition state. The later advantage is fully operational in modern biology because of the evolution of highly effective and selective enzymes. However, it constituted very probably a strong limitation in chemical systems having limited possibilities of selective catalysis. Therefore, the early role of phosphate-derived species is more likely to be the result of their lack of reactivity than that of possibilities of transferring energy between metabolic subsystems. We therefore conclude that the role of ATP as a universal energy currency is unlikely to be an early invention of life. In spite of these limitations, it is possible to depict the possibilities opened by phosphate chemistry at an early stage just by considering its specific reactivity. Though their role could be limited to specific processes, mixed anhydrides could have played a role in transferring energy from the chemistry of amino acids to that of nucleotides being essential in the emergence of translation. More generally, pathways for the phosphorylation of nucleosides and hydrophobic alcohols are available in an origin of life context. As mentioned above, a very important property of phosphate derivatives such as phosphate esters is their reduced kinetic reactivity. This property has certainly been selected for information storage and is a major reason for the selection of the phosphodiester-based nucleic acid backbone, which is expressed at the highest degree in DNA. It could additionally be considered that the lack of reactivity of phosphate esters is also revealed by the difficulty in building the phosphodiester bond. Imidazolides and their derivatives have been considered in many RNA world experiments as convenient activated monomers for RNA polymerization [115] rather than the biochemical triphosphate substrates. This possibility is supported by new reports on the relevance of the abiotic synthesis of imidazole derivatives under early Earth conditions as well as to their specific reactivity [31,116]. 

## Figures and Tables

**Table 1:** Values of the free energy of hydrolysis at pH 7 for different phosphate-based energy-rich biochemical metabolites.

Reagent	Product(s)	Δ*G*°’ kJ mol^−1^	Reference
PPi	2 Pi	−19	[33]
ATP	AMP + PPi	−32.2	[33]
ATP	ADP + Pi	−30.5	[33]
Acetyl phosphate	AcOH + Pi	−43.1	[33]
Carbamyl phosphate	CO_2_ + NH_3_ + Pi	ca. −51 ^1^	[33]
Aminoacyl phosphate	Amino acid + Pi	ca. −50	[91]
Aminoacyl adenylate	Amino acid + AMP	−70	[44]
Phosphoenol pyruvate	Pyruvate + Pi	−62	[33]

^1^ Value determined at pH 9.5.

**Table 2:** Values of the free energy of hydrolysis at pH 7 for different potential phosphate activating agents available in the literature.

Reagent	Product(s)	Δ*G*°’ kJ mol^−1^	Reference
HNCO	CO_2_ + NH_3_	−54	[91]
Urea	CO_2_ + NH_3_	−28	[91]
Cyanamide	Isourea	−83	[94]
Carbodiimide	Isourea	−97	[94]
Acetic anhydride	Acetic acid	−91	[33]
NCA	Amino acid + CO_2_	−60	[46]

## References

[1] Lipmann F. (1941). Metabolic generation and utilization of phosphate bond energy. Adv. Enzymol. Relat. Areas Mol. Biol..

[2] Lipmann F., McElroy W.D., Glass H.B. (1951). Phosphorus Metabolism.

[3] Westheimer F.H. (1987). Why nature chose phosphate. Science.

[4] Kumler W.D., Eiler J.J. (1943). The Acid Strength of Mono and Diesters of Phosphoric Acid. The n-Alkyl Esters from Methyl to Butyl, the Esters of Biological Importance, and the Natural Guanidine Phosphoric Acids. J. Am. Chem. Soc..

[5] Wolfenden R., Ridgway C., Young G. (1998). Spontaneous hydrolysis of ionized phosphate monoesters and diesters and the proficiencies of phosphatases and phosphodiesterases as catalysts. J. Am. Chem. Soc..

[6] Bowler M.W., Cliff M.J., Waltho J.P., Blackburn G.M. (2010). Why did Nature select phosphate for its dominant roles in biology?. New J. Chem..

[7] Goldford J.E., Hartman H., Smith T.F., Segrè D. (2017). Remnants of an Ancient Metabolism without Phosphate. Cell.

[8] Martin W.F., Thauer R.K. (2017). Energy in Ancient Metabolism. Cell.

[9] Pasek M.A., Kee T.P., Bryant D.E., Pavlov A.A., Lunine J.I. (2008). Production of Potentially Prebiotic Condensed Phosphates by Phosphorus Redox Chemistry. Angew. Chem. Int. Ed..

[10] Bryant D.E., Greenfield D., Walshaw R.D., Evans S.M., Nimmo A.E., Smith C.L., Wang L., Pasek M.A., Kee T.P. (2009). Electrochemical studies of iron meteorites: Phosphorus redox chemistry on the early Earth. Int. J. Astrobiol..

[11] Bryant D.E., Marriott K.E.R., Macgregor S.A., Kilner C., Pasek M.A., Kee T.P. (2010). On the prebiotic potential of reduced oxidation state phosphorus: The H-phosphinate–pyruvate system. Chem. Commun..

[12] Kaye K., Bryant D., Marriott K., Ohara S., Fishwick C., Kee T. (2016). Selective Phosphonylation of 5′-Adenosine Monophosphate (5′-AMP) via Pyrophosphite [PPi(III)]. Orig. Life Evol. Biosph..

[13] Schroeder G.K., Lad C., Wyman P., Williams N.H., Wolfenden R. (2006). The time required for water attack at the phosphorus atom of simple phosphodiesters and of DNA. Proc. Natl. Acad. Sci. USA.

[14] Wolfenden R. (2011). Benchmark reaction rates, the stability of biological molecules in water, and the evolution of catalytic power in enzymes. Annu. Rev. Biochem..

[15] Kirby A.J. (1980). Effective molarities for intramolecular reactions. Adv. Phys. Org. Chem..

[16] Pascal R. (2003). Catalysis through Induced Intramolecularity: What Can Be Learned by Mimicking Enzymes with Carbonyl Compounds that Covalently Bind Substrates?. Eur. J. Org. Chem..

[17] Pascal R. (2015). Kinetic Barriers and the Self-organization of Life. Isr. J. Chem..

[18] Li B.-J., El-Nachef C., Beauchemin A. (2017). Organocatalysis Using Aldehydes: The Development and Improvement of Catalytic Hydroaminations, Hydrations and Hydrolyses. Chem. Commun..

[19] Kirby A.J., Varvoglis A.G. (1967). The Reactivity of Phosphate Esters. Monoester Hydrolysis. J. Am. Chem. Soc..

[20] Westheimer F.H. (1981). Monomeric metaphosphate ion. Chem. Rev..

[21] Kamerlin S.C., Sharma P.K., Prasad R.B., Warshel A. (2013). Why nature really chose phosphate. Q. Rev. Biophys..

[22] Lipmann F., Fox S.W. (1965). Projecting backward from the present stage of evolution of biosynthesis. The Origins of Prebiological Systems and of Their Molecular Matrices.

[23] Miller S.L., Parris M. (1964). Synthesis of pyrophosphate under primitive Earth conditions. Nature.

[24] Vieyra A., Meyer-Fernandes J.R., Gama O.B. (1985). Phosphorolysis of acetyl phosphate by orthophosphate with energy conservation in the phosphoanhydride linkage of pyrophosphate. Arch. Biochem. Biophys..

[25] Vieyra A., Gueiros-Filho F., Meyer-Fernandes J.R., Costa-Sarmento G., De Souza-Barros F. (1995). Reactions involving carbamyl phosphate in the presence of precipitated calcium phosphate with formation of pyrophosphate: A model for primitive energy-conservation pathways. Orig. Life Evol. Biosph..

[26] Keefe A.D., Miller S.L. (1995). Are polyphosphate or phosphate esters prebiotic reagents?. J. Mol. Evol..

[27] Keefe A.D., Miller S.L. (1996). Potentially prebiotic synthesis of condensed phosphates. Orig. Life Evol. Biosph..

[28] Deamer D.W. (1997). The First Living Systems: A Bioenergetic Perspective. Microbiol. Mol. Biol. Rev..

[29] Russell M.J. (2007). The Alkaline Solution to the Emergence of Life: Energy, Entropy and Early Evolution. Acta Biotheor..

[30] Hagan W.J., Parker A., Steuerwald A., Hathaway M. (2007). Phosphate Solubility and the Cyanate-Mediated Synthesis of Pyrophosphate. Orig. Life Evol. Biosph..

[31] Szostak J. (2017). The Origin of Life on Earth and the Design of Alternative Life Forms. Mol. Front. J..

[32] Lipmann F. (1946). Acetyl Phosphate. Adv. Enzymol. Relat. Areas Mol. Biol..

[33] Jencks W.P., Fasman G.D. (1976). Free energies of hydrolysis and decarboxylation. Handbook of Biochemistry and Molecular Biology.

[34] Di Sabato G., Jencks W.P. (1961). Mechanism and catalysis of reactions of acyl phosphates II. Hydrolysis. J. Am. Chem. Soc..

[35] Biron J.-P., Pascal R. (2004). Amino acid *N*-carboxyanhydrides: Activated peptide monomers behaving as phosphate-activating agents in aqueous solution. J. Am. Chem. Soc..

[36] O’Connor C.J., Wallace R.G. (1984). A phosphate-catalysed acyl transfer reaction. Hydrolysis of 4-nitrophenyl acetate in phosphate buffers. Aust. J. Chem..

[37] Andrés G.O., Granados A.M., de Rossi R.H. (2001). Kinetic Study of the Hydrolysis of Phthalic Anhydride and Aryl Hydrogen Phthalates. J. Org. Chem..

[38] El Seoud O.A., Ruasse M.-F., Rodrigues W.A. (2002). Kinetics and mechanism of phosphate-catalyzed hydrolysis of benzoate esters: Comparison with nucleophilic catalysis by imidazole and *o*-iodosobenzoate. J. Chem. Soc. Perkin Trans. 2.

[39] Gill M.S., Neverov A.A., Brown R.S. (1997). Dissection of nucleophilic and general base roles for the reaction of phosphate with *p*-nitrophenylthiolacetate, *p*-nitrophenylthiolformate, phenylthiolacetate. J. Org. Chem..

[40] Thomas G.L., Payne R.J. (2009). Phosphate-assisted peptide ligation. Chem. Commun..

[41] Higuchi T., Flynn G.L., Shah A.C. (1967). Reversible Formation and Hydrolysis of Phthaloyl and Succinyl Monophosphates in Aqueous Solution. J. Am. Chem. Soc..

[42] Hagan W.J. (2010). Uracil-Catalyzed Synthesis of Acetyl Phosphate: A Photochemical Driver for Protometabolism. ChemBioChem.

[43] Whicher A., Camprubi E., Pinna S., Herschy B., Lane N. (2018). Acetyl Phosphate as a Primordial Energy Currency at the Origin of Life. Orig. Life Evol. Biosph..

[44] Wells T.N.C., Ho C.K., Fersht A.R. (1986). Free Energy of Hydrolysis of Tyrosyl Adenylate and Its Binding to Wild-Type and Engineered Mutant Tyrosyl-tRNA Synthetases. Biochemistry.

[45] Ribas de Pouplana L., Schimmel P. (2001). Aminoacyl-tRNA synthetases: Potential markers of genetic code development. Trends Biochem. Sci..

[46] Pascal R., Boiteau L., Commeyras A. (2005). From the Prebiotic Synthesis of α-Amino Acids Towards a Primitive Translation Apparatus for the Synthesis of Peptides. Top. Curr. Chem..

[47] Pascal R., Boiteau L., Gargaud M., Lopez-Garcia P., Martin H. (2011). Energetic constraints on prebiotic pathways: Application to the emergence of translation. Origin and Evolution of Life: An Astrobiology Perspective.

[48] Liu Z., Beaufils D., Rossi J.-C., Pascal R. (2014). Evolutionary importance of the intramolecular pathways of hydrolysis of phosphate ester mixed anhydrides with amino acids and peptides. Sci. Rep..

[49] Liu Z., Rigger L., Rossi J.-C., Sutherland J.D., Pascal R. (2016). Mixed Anhydride Intermediates in the Reaction of 5(4*H*)-Oxazolones with Phosphate Esters and Nucleotides. Chem. Eur. J..

[50] Biron J.-P., Parkes A.L., Pascal R., Sutherland J.D. (2005). Expeditious, potentially primordial, aminoacylation of nucleotides. Angew. Chem. Int. Ed. Engl..

[51] Leman L., Orgel L., Ghadiri M.R. (2006). Amino Acid Dependent Formation of Phosphate Anhydrides in Water Mediated by Carbonyl Sulfide. J. Am. Chem. Soc..

[52] Brack A. (1987). Selective emergence and survival of early polypeptides in water. Orig. Life.

[53] Commeyras A., Taillades J., Collet H., Boiteau L., Vandenabeele-Trambouze O., Pascal R., Rousset A., Garrel L., Rossi J.-C., Biron J.-P. (2004). Dynamic co-evolution of peptides and chemical energetics, a gateway to the emergence of homochirality and the catalytic activity of peptides. Orig. Life Evol. Biosph..

[54] Leman L., Orgel L., Ghadiri M.R. (2004). Carbonyl Sulfide–Mediated Prebiotic Formation of Peptides. Science.

[55] Danger G., Boiteau L., Cottet H., Pascal R. (2006). The peptide formation mediated by cyanate revisited. N-carboxyanhydrides as accessible intermediates in the decomposition of N-carbamoylamino acids. J. Am. Chem. Soc..

[56] Wickramasinghe N.S.M.D., Staves M.P., Lacey J.C. (1991). Stereoselective, nonenzymatic, intramolecular transfer of amino acids. Biochemistry.

[57] Paecht-Horowitz M., Berger J., Katchalsky A. (1970). Prebiotic synthesis of polypeptides by heterogeneous polycondensation of aminoacid adenylates. Nature.

[58] Liu Z., Hanson C., Ajram G., Boiteau L., Rossi J.-C., Danger G., Pascal R. (2017). 5(4*H*)-Oxazolones as Effective Aminoacylation Reagents for the 3′-Terminus of RNA. Synlett.

[59] Bowler F.R., Chan C.K., Duffy C.D., Gerland B., Islam S., Powner M.W., Sutherland J.D., Xu J. (2013). Prebiotically plausible oligoribonucleotide ligation facilitated by chemoselective acetylation. Nat. Chem..

[60] Liu Z., Ajram G., Rossi J.-C., Pascal R. (2019). The chemical likelihood of ribonucleotide-α-amino acid copolymers as players for early stages of evolution. J. Mol. Evol..

[61] Page M.I., Jencks W.P. (1971). Entropic contribution to rate accelerations in enzymic and intramolecular reactions and the chelate effect. Proc. Natl. Acad. Sci. USA.

[62] De Duve C. (2003). A research proposal on the origin of life. Orig. Life Evol. Biosph..

[63] Adelfinskaya O., Herdewijn P. (2007). Amino Acid Phosphoramidate Nucleotides as Alternative Substrates for HIV-1 Reverse Transcriptase. Angew. Chem. Int. Ed..

[64] Adelfinskaya O., Terrazas M., Froeyen M., Marlière P., Nauwelaerts K., Herdewijn P. (2007). Polymerase-catalyzed synthesis of DNA from phosphoramidate conjugates of deoxynucleotides and amino acids. Nucleic Acids Res..

[65] Giraut A., Herdewijn P. (2010). Influence of the Linkage between Leaving Group and Nucleoside on Substrate Efficiency for Incorporation in DNA Catalyzed by Reverse Transcriptase. ChemBioChem.

[66] Song X.-P., Bouillon C., Lescrinier E., Herdewijn P. (2011). Iminodipropionic acid as the leaving group for DNA polymerization by HIV-1 reverse transcriptase. ChemBioChem.

[67] Song X.-P., Bouillon C., Lescrinier E., Herdewijn P. (2012). Dipeptides as Leaving Group in the Enzyme-Catalyzed DNA Synthesis. Chem. Biodivers..

[68] Giraut A., Abu El-Asrar R., Marlière P., Delarue M., Herdewijn P. (2012). 2′-Deoxyribonucleoside phosphoramidate triphosphate analogues as alternative substrates for E. coli polymerase III. ChemBioChem.

[69] Cheng C.M., Liu X.H., Li Y.M., Ma Y., Tan B., Wan R., Zhao Y.F. (2004). *N*-Phosphoryl amino acids and biomolecular origins. Orig. Life Evol. Biosph..

[70] Gao X., Deng H., Tang G., Liu Y., Xu P., Zhao Y. (2011). Intermolecular Phosphoryl Transfer of *N*-Phosphoryl Amino Acids. Eur. J. Org. Chem..

[71] Ying J., Fu S., Li X., Fen L., Pengxiang X., Liu Y., Gao X., Zhao Y. (2018). A plausible model correlates prebiotic peptide synthesis with the primordial genetic code. Chem. Commun..

[72] Griesser H., Tremmel P., Kervio E., Pfeffer C., Steiner U.E., Richert C. (2017). Ribonucleotides and RNA Promote Peptide Chain Growth. Angew. Chem. Int. Ed..

[73] Jauker M., Griesser H., Richert C. (2015). Spontaneous Formation of RNA Strands, Peptidyl RNA, and Cofactors. Angew. Chem. Int. Ed..

[74] Krishnamurthy R., Arrhenius G., Eschenmoser A. (1999). Formation of glycolaldehyde phosphate from glycolaldehyde in aqueous solution. Orig. Life Evol. Biosph..

[75] Krishnamurthy R., Guntha S., Eschenmoser A. (2000). Regioselective α-phosphorylation of aldoses in aqueous solution. Angew. Chem. Int. Ed..

[76] Karki M., Gibard C., Bhowmik S., Krishnamurthy R. (2017). Nitrogenous Derivatives of Phosphorus and the Origins of Life: Plausible Prebiotic Phosphorylating Agents in Water. Life.

[77] Gibard C., Bhowmik S., Karki M., Kim E.-K., Krishnamurthy R. (2017). Phosphorylation, oligomerization and self-assembly in water under potential prebiotic conditions. Nat. Chem..

[78] Lohrmann R., Orgel L.E. (1976). Template-directed synthesis of high molecular weight polynucleotide analogues. Nature.

[79] Zielinski W.S., Orgel L.E. (1985). Oligomerization of activated derivatives of 3′-amino-3′-deoxyguanosine on poly(C) and poly(dC) templates. Nucleic Acids Res..

[80] Zielinski W.S., Orgel L.E. (1987). Autocatalytic synthesis of a tetranucleotide analogue. Nature.

[81] Zielinski W.S., Orgel L.E. (1987). Oligoaminonucleoside phosphoramidates. Oligomerization of dimers of 3′-amino-3′-deoxynudeotides (GC and CG) in aqueous solution. Nucleic Acids Res..

[82] Zielinski W.S., Orgel L.E. (1989). Polymerization of a Monomeric Guanosine Derivative in a Hydrogen-Bonded Aggregate. J. Mol. Evol..

[83] Sievers D., von Kiedrowski G. (1994). Self-replication of complementary nucleotide-based oligomers. Nature.

[84] Mansy S.S., Schrum J.P., Krishnamurthy M., Tobé S., Treco D.A., Szostak J.W. (2008). Template-directed synthesis of a genetic polymer in a model protocell. Nature.

[85] Zhang S., Blain C., Zielinska D., Gryaznov S., Szostak J. (2013). Fast and accurate nonenzymatic copying of an RNA-like synthetic genetic polymer. Proc. Natl. Acad. Sci. USA.

[86] Shim J.L., Lohrmann R., Orgel L.E. (1974). Poly(U)-Directed Transamidation between Adenosine 5′-Phosphorimidazolide and 5′ Phosphoadenosine 2′(3′)-Glycine Ester. J. Am. Chem. Soc..

[87] Nicolis G., Prigogine I. (1977). Self-Organization in Nonequilibrium Systems: From Dissipative Structures to Order through Fluctuations.

[88] Eschenmoser A. (1994). Chemistry of potentially prebiological natural products. Orig. Life Evol. Biosph..

[89] Eschenmoser A. (2007). Question 1: Commentary Referring to the Statement “The Origin of Life can be Traced Back to the Origin of Kinetic Control” and the Question “Do You Agree with this Statement; and How Would You Envisage the Prebiotic Evolutionary Bridge Between Thermodynamic and Kinetic Control?” Stated in Section 1.1. Orig. Life Evol. Biosph..

[90] Danger G., Plasson R., Pascal R. (2012). Pathways for the formation and evolution of peptides in prebiotic environments. Chem. Soc. Rev..

[91] Boiteau L., Pascal R. (2011). Energy sources, self-organization, and the origin of life. Orig. Life Evol. Biosph..

[92] Schwartz A.W. (2006). Phosphorus in prebiotic chemistry. Philos. Trans. R. Soc. Lond. Ser. B.

[93] Fernández-García C., Coggins A.J., Powner M.W. (2017). A Chemist’s Perspective on the Role of Phosphorus at the Origins of Life. Life.

[94] Tordini F., Bencini A., Bruschi M., De Gioia L., Zampella G., Fantucci P. (2003). Theoretical Study of Hydration of Cyanamide and Carbodiimide. J. Phys. Chem. A.

[95] Steinman G., Lemmon R.M., Calvin M. (1964). Cyanamide: A possible key compound in chemical evolution. Proc. Natl. Acad. Sci. USA.

[96] Jones M.E., Spector L., Lipmann F. (1955). Carbamyl phosphate, the carbamyl donor in enzymatic citrulline synthesis. J. Am. Chem. Soc..

[97] Jones M.E., Lipmann F. (1960). Chemical and enzymatic synthesis of carbamyl phosphate. Proc. Natl. Acad. Sci. USA.

[98] Halmann M., Lapidot A., Samuel D. (1962). Kinetic and tracer studies of the reaction of carbamoyl phosphate in aqueous solution. J. Chem. Soc..

[99] Allen C.M., Jones M.E. (1964). Decomposition of Carbamylphosphate in Aqueous Solutions. Biochemistry.

[100] Vogels G.D., Uffink L., Van der Drift C. (1970). Cyanate decomposition catalyzed by certain divalent anions. Recl. Trav. Chim. Pays-Bas.

[101] Danger G., Charlot S., Boiteau L., Pascal R. (2012). Activation of carboxyl group with cyanate: Peptide bond formation from dicarboxylic acids. Amino Acids.

[102] Saygin Ö. (1981). Non-enzymatic photophosphorylation with visible-light—A possible mode of prebiotic ATP. Naturwissenschaften.

[103] Saygin Ö. (1983). Non-enzymatic phosphorylation of acetate by carbamyl-phosphate—A model reaction for prebiotic activation of carboxyl groups. Orig. Life.

[104] Saygin Ö. (1984). Photochemical carbamylphosphate formation and metal ion-catalysed transphosphorylations between carbamylphosphate and adenine nucleotides or carboxyl groups. Orig. Life.

[105] Hosseini M.W., Lehn J.M. (1987). Supramolecular catalysis of phosphoryl transfer: Pyrophosphate synthesis from acetyl phosphate mediated by macrocyclic polyamines. J. Am. Chem. Soc..

[106] Hosseini M.W., Lehn J.M. (1991). Supramolecular catalysis of adenosine triphosphate synthesis in aqueous solution mediated by a macrocyclic polyamine and divalent metal cations. J. Chem. Soc. Chem. Commun..

[107] Costanzo G., Saladino R., Crestini C., Ciciriello F., Di Mauro E. (2007). Nucleoside Phosphorylation by Phosphate Minerals. J. Biol. Chem..

[108] Lohrmann R., Orgel L.E. (1971). Urea-Inorganic Phosphate Mixtures as Prebiotic Phosphorylating Agents. Science.

[109] Reimann R., Zubay G. (1999). Nucleoside phosphorylation: A feasible step in the prebiotic pathway to RNA. Orig. Life Evol. Biosph..

[110] Albertsen A., Duffy C., Sutherland J., Monnard P. (2014). Self-Assembly of Phosphate Amphiphiles in Mixtures of Prebiotically Plausible Surfactants. Astrobiology.

[111] Orgel L.E., Lohrmann R. (1974). Prebiotic Chemistry and Nucleic Acid replication. Acc. Chem. Res..

[112] Osterberg R., Orgel L.E. (1972). Polyphosphate and Trimetaphosphate Formation under Potentially Prebiotic Conditions. J. Mol. Evol..

[113] Fiore M., Madanamoothoo W., Berlioz-Barbier A., Maniti O., Girard-Egrot A., Buchet R., Strazewski P. (2017). Giant vesicles from rehydrated crude mixtures containing unexpected mixtures of amphiphiles formed under plausibly prebiotic conditions. Org. Biomol. Chem..

[114] Shaw W.H.R., Bordeaux J.J. (1955). The Decomposition of Urea in Aqueous Media. J. Am. Chem. Soc..

[115] Orgel L.E. (2004). Prebiotic chemistry and the origin of the RNA world. Crit. Rev. Biochem. Mol. Biol..

[116] Fahrenbach A.C., Giurgiu C., Tam C.P., Li L., Hongo Y., Aono M., Szostak J.W. (2017). Common and Potentially Prebiotic Origin for Precursors of Nucleotide Synthesis and Activation. J. Am. Chem. Soc..

